# Novel *Rickettsia raoultii* strain isolated and propagated from Austrian *Dermacentor reticulatus* ticks

**DOI:** 10.1186/s13071-016-1858-x

**Published:** 2016-11-03

**Authors:** Michiel Wijnveld, Anna-Margarita Schötta, Adriano Pintér, Hannes Stockinger, Gerold Stanek

**Affiliations:** 1 Institute for Hygiene and Applied Immunology, Center for Pathophysiology, Infectiology and Immunology, Medical University of Vienna, Kinderspitalgasse 15, 1090 Vienna, Austria; 2 Public Health Department, Superintendência de Controle de Endemias, Rua Paula Sousa, 166 - Luz - 01027-000, São Paulo, São Paulo Brazil

**Keywords:** *Rickettsia raoultii*, *Dermacentor reticulatus*, Austria, BME/CTVM2, Isolation, Culture

## Abstract

**Background:**

Continuous culture of tick cell lines has proven a valuable asset in isolating and propagating several different vector-borne pathogens, making it possible to study these microorganisms under laboratory conditions and develop serological tests to benefit public health. We describe a method for effective, cost- and labor-efficient isolation and propagation of *Rickettsia raoultii* using generally available laboratory equipment and *Rhipicephalus microplus* cells, further demonstrating the usefulness of continuous tick cell lines. *R. raoultii* is one of the causative agents of tick-borne lymphadenopathy (TIBOLA) and is, together with its vector *Dermacentor reticulatus*, emerging in novel regions of Europe, giving rise to an increased threat to general public health.

**Methods:**

*Dermacentor reticulatus* ticks were collected in the Donau-Auen (Lobau) national park in Vienna, Austria. The hemolymph of ten collected ticks was screened by PCR-reverse line blot for the presence of rickettsial DNA. A single tick tested positive for *R. raoultii* DNA and was used to infect *Rhipicephalus microplus* BME/CTVM2 cells.

**Results:**

Sixty-five days after infection of the tick-cell line with an extract from a *R. raoultii-*infected tick, we observed intracellular bacteria in the cultured cells. On the basis of microscopy we suspected that the intracellular bacteria were a species of *Rickettsia*; this was confirmed by several PCRs targeting different genes. Subsequent sequencing showed 99–100 % identity with *R. raoultii*. Cryopreservation and resuscitation of *R. raoultii* was successful. After 28 days identical intracellular bacteria were microscopically observed.

**Conclusions:**

*R. raoultii* was successfully isolated and propagated from *D. reticulatus* ticks using *R. microplus* BME/CTVM2 cells. The isolated strain shows significant molecular variation compared to currently known sequences. Furthermore we show for the first time the successful cryopreservation and resuscitation of *R. raoultii*.

## Background

**Fig. 1:**
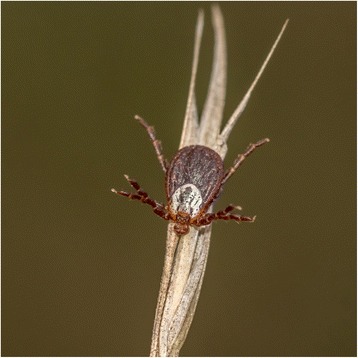
Female *Dermacentor reticulatus* tick in a questing position

*Dermacentor reticulatus*, also known as the ornate dog tick, is a dark-colored tick with a light-colored pattern on its scutum (Fig. 1). Recorded hosts of *D. reticulatus* include wild and domesticated carnivores, sheep, cattle, and horses [1, 2]; human infestation is not uncommon [2–4].

The typical habitats of this tick are open areas such as meadows, dune valleys, and floodplains [2] with a high degree of humidity [1]; this is in contrast to *Ixodes ricinus*, which prefers greater shelter within deciduous and coniferous woodland areas [1]. In addition, the seasonal activity of *D. reticulatus* differs from that of most other European tick species, with larvae and nymphs being active in spring and summer, followed by adult activity starting in early autumn going late into winter, with a short pause when conditions become too harsh, activity then resuming thereafter leading in several geographical locations to an additional activity peak in spring [1, 2, 5–7]. This pattern of activity results in *D. reticulatus* being active for nearly the whole year. Furthermore, it has become apparent in recent years that *D. reticulatus* is spreading to new areas, increasing its foothold within Europe [2, 8–12] and increasing exposure of humans and animals to this tick species and its transmitted pathogens. Thus, the expansion of *D. reticulatus* into new territories, with seasonal activity in northern regions unlike that of *I. ricinus*, and the possible transmission of zoonotic pathogens pose a serious risk. Public awareness of the dangers of *D. reticulatus* bites needs to increase.

Among the pathogens transmitted by *D. reticulatus*, *Rickettsia raoultii* has a primary position, with infection rates up to 20 % in questing ticks [13], in some areas reaching even higher rates (50–58 %) [14, 15]. *Rickettsia raoultii* belongs to the spotted fever group *Rickettsia* and is one of the causative agents of tick-borne lymphadenopathy (TIBOLA), which is also known as *Dermacentor*-borne necrosis erythema and lymphadenopathy (DEBONEL) in humans [3]. Symptoms include an inoculation eschar at the tick-bite location, (cervical) lymphadenopathy, (high) fever, malaise, and headaches [16]. As its vector expands to new foci, with recent novel detections in several countries of Europe, *R. raoultii* presents as an emerging disease-causing agent [17–20].

The increasing medical relevance of *R. raoultii* in Europe requires further studies of this organism. Here we describe a method for its isolation and propagation using generally available laboratory equipment and the low-maintenance embryo-derived tick cell line BME/CTVM2 derived from *Rhipicephalus microplus* [21].

## Methods

### Ticks

*Dermacentor reticulatus* ticks were visualized and collected directly from the vegetation in the Donau-Auen (Lobau) national park in Vienna, Austria, in October 2015, and were morphologically identified using standard identification keys [1]. Ten of the collected ticks were selected randomly to screen for the presence of *Rickettsia raoultii* DNA.

### DNA extraction from hemolymph of single tick legs

A single leg was cut from each of the ten selected ticks; the ticks were kept alive in individual collection tubes stored at 4 °C until further use. DNA was extracted from individual tick legs using the NucleoSpin Tissue XS kit (Macherey-Nagel, Düren, Germany) according to the manufacturer’s instructions and with a total elution volume of 15 μl.

### PCR-reverse line blot

Rickettsial DNA was detected using PCR followed by reverse line blot (RLB) hybridization targeting the *Rickettsia* spp. 16S rRNA gene, as previously described [22, 23]. *Rickettsia* genus-specific and *R. conorii* and *R. raoultii* species-specific probes described by Christova et al. and Nijhof et al. were used [23, 24]. Briefly, the PCR reaction mix (total reaction volume 25 μl) contained 5 μl (5×) Phire reaction buffer, 200 nmol/l of each dNTP (Solis Biodyne, Tartu, Estonia), 400 nmol/l of each primer (Rick-F1 and Rick-R2 as in Table 1), 0.125 units Phire Hot Start II DNA Polymerase (Thermo Scientific, Vienna, Austria), 2.5 μl template DNA [DNA extracts, *R. conorii* positive control (Ingenetix, Vienna, Austria), and no-template control, respectively], and PCR-grade water (Sigma-Aldrich, Vienna, Austria). A C1000 Touch Thermal Cycler (Bio-Rad, Vienna, Austria) was used for the PCR reactions, starting with a touch-down protocol in which the annealing temperature was lowered by 1° per cycle for the initial 10 cycles (98 °C for 5 s, 67 °C to 57 °C for 5 s, and 72 °C for 10 s) followed by 45 cycles with a fixed annealing temperature (98 °C for 5 s, 57 °C for 5 s, and 72 °C for 10 s). The initial touch-down cycles were used to increase the specificity of the PCR reactions. All PCR products were used in the RLB assay as described previously [25] and were visualized using the ChemiDoc Touch Imaging System (Bio-Rad). The RLB membrane was exposed for 10 min to detect the chemiluminescence of the bound PCR products.

**Table 1:** Primers used during this study

Name	Sequence (5′-3′)	Annealing temperature (°C)	Target	Reference
Rick-F1	GAACGCTATCGGTATGCTTAACACA	67–57^a^	16S ribosomal RNA gene	[23]
Rick-R2	CATCACTCACTCGGTATTGCTGGA			
RCK/23-5-F	GATAGGTCRGRTGTGGAAGCAC	65–55^a^	23S–5S ribosomal RNA intergenic spacer	[29]
RCK/23-5-R	TCGGGAYGGGATCGTGTGTTTC			
D1f	ATGAGTAAAGACGGTAACCT	60	*sca4*	[37]
D928r	AAGCTATTGCGTCATCTCCG			
190-70	ATGGCGAATATTTCTCCAAAA	60	*ompA*	[38]
190-701	GTTCCGTTAATGGCAGCATCT			
120-2788	AAACAATAATCAAGGTACTGT	60	*ompB*	[39]
120-3599	TACTTCCGGTTACAGCAAAGT			
CS-78	GCAAGTATCGGTGAGGATGTAAT	60	*gltA*	[40]
CS-323	GCTTCCTTAAAATTCAATAAATCAGGAT			

^a^ Touch down PCR protocol decreasing annealing temperature by 1 °C per cycle during the first 10 cycles

### Tick surface disinfection and homogenization

A single tick from the ten that were tested gave a positive result for presence of *R. raoultii* DNA. The surface of the tick was disinfected using thiomersal, benzalkonium chloride and 70 % ethanol solutions followed by 2 rinses in sterile demineralized water, as described by Bell-Sakyi [26]. After disinfection, the tick was cut with a sterile scalpel and homogenized in complete L-15 cell culture medium (Leibovitz’s L-15 medium, Gibco, Thermofisher Scientific, Vienna, Austria) [27] using a 100–1,000 μl Radnoti Micro Homogenizer (Radnoti, Dublin, Ireland). The cell suspension in the homogenate was separated from tick debris using a 1 ml Micro-Fine™ insulin syringe, 29 gauge (BD, Oxford, UK). Portions (100 μl) of the obtained cell suspension were transferred directly into two individual tick cell cultures; a third tick cell culture was maintained as a negative control. All 3 cell cultures originated from a single parental culture to eliminate cell variation. All steps were performed under sterile conditions in a class II laminar flow cabinet.

### Culture of tick cell line

The tick cell line BME/CTVM2 (The Tick Cell Biobank, Pirbright, UK) originating from *R. microplus* [21] was maintained, as previously described [27], at 28 °C in flat-sided cell culture tubes (Nunc, Thermofisher Scientific, Vienna, Austria) containing 2 ml L-15 medium supplemented with 2 mmol/L L-glutamine (HyClone, GE Healthcare Life sciences, Vienna, Austria), 10 % tryptose phosphate broth (Gibco, Thermofisher Scientific, Vienna, Austria), 20 % fetal calf serum (Sigma Aldrich, Vienna, Austria), and 100 U/ml penicillin and 100 μg/ml streptomycin (Pen Strep, Gibco). The medium was changed weekly, when samples of the infected and the negative-control cell cultures were taken for cytological analysis.

### Staining of cytocentrifuge smears

After the weekly change of medium, tick cells were resuspended using a glass Pasteur pipette. From this suspension ≈ 100 μl was used to prepare cytocentrifuge smears using a Basic Cytology Rotor in a Rotofix 32A centrifuge (Hettich Lab Technology, Tuttlingen, Germany) with a single cytology funnel (Biomedical Polymers, Gardner, MA, USA). Centrifugation conditions were 700× *g* for 5 min. The obtained smears were stained with Giménez stain [28]. Stained smears were analyzed microscopically for the presence of *Rickettsia*-like intracellular bacteria.

### DNA extraction of tick cell cultures, PCR and sequencing

After observing *Rickettsia*-like intracellular bacteria, DNA was extracted from 200 μl cell suspension using the NucleoSpin Tissue kit (Macherey-Nagel, Düren, Germany) according to the manufacturer’s guidelines. The presence and identity of suspected rickettsial bacteria was confirmed using several PCRs targeting different genes and regions: *gltA*, *ompA*, *ompB*, *sca4*, 16S ribosomal RNA, and the 23S-5S ribosomal RNA intergenic spacer (see Table 1 for primers and annealing temperatures). Phire Hot Start II DNA Polymerase (0.5 units) and a reduced volume of PCR-grade water were used. The concentration of the other components in the PCR mix were as described above. PCR products were analyzed using 1 % agarose gel electrophoresis, loading the full 25 μl of PCR product onto the gel. Subsequently, fragments of interest were extracted from the gel using the QIAquick gel extraction kit (Qiagen, Hilden, Germany), following the protocol provided. All obtained PCR fragments were sent to Microsynth (Balgach, Switzerland) for bidirectional sequencing, and consensus sequences have been submitted to GenBank (http://www.ncbi.nlm.nih.gov/genbank/) under accession numbers KX500092–KX500097.

### PCR-RLB detection of *R. raoultii* strain Jongejan

To detect the isolated *R. raoultii* strain, a new strain-specific probe was designed (*R. raoultii* strain Jongejan probe: 5′-TCA ACT AAT AAA TTT GCT GAG TA-3′) to be used in a PCR-RLB assay targeting the 23S-5S ribosomal RNA intergenic spacer [29]. The procedure was performed as described above, with some minor modifications. Briefly, a touch-down protocol was used in the PCR (65 °C going down to 55 °C during the first ten cycles) and in the RLB the primers and probes were as described by Jado et al. [29], together with the newly designed strain-specific probe.

### Cryopreservation and resuscitation of *Rickettsia raoultii*

Combining and modifying previously published methods [30, 31], cryopreservation and resuscitation was carried out as follows: infected cell suspensions were transferred to 2 ml safe-lock microcentrifuge tubes (Eppendorf AG, Hamburg, Germany), and chilled on ice before centrifugation. Centrifugation was carried out at 17,000× g for 10 min at 4 °C. After discarding the supernatant, the pellet was resuspended using half the original volume of fresh complete L-15 medium. After resuspension, complete L-15 medium supplemented with 20 % dimethylsulfoxide (DMSO) was added until 100 % of the original volume was reached, obtaining a final DMSO concentration of 10 %. All components were used pre-chilled at 4 °C. Aliquots (100 μl) were prepared in CryoTubes (Nunc, Thermofisher Scientific, Vienna, Austria) and flash frozen in liquid nitrogen; aliquots were stored either at -80 °C or in liquid nitrogen. Resuscitation was carried out by rapidly thawing the cell suspensions in a water bath at 37 °C, adding 1.9 ml complete L-15 medium, and transferring the suspension to 2 ml safe-lock microcentrifuge tubes. Subsequently, the cell suspensions were centrifuged at 17,000× g for 10 min at 4 °C, supernatant discarded and the pellet was resuspended in 100 μl complete L-15 medium. The cell suspension was directly used to inoculate uninfected BME/CTVM2 tick cells.

## Results

**Fig. 2:**
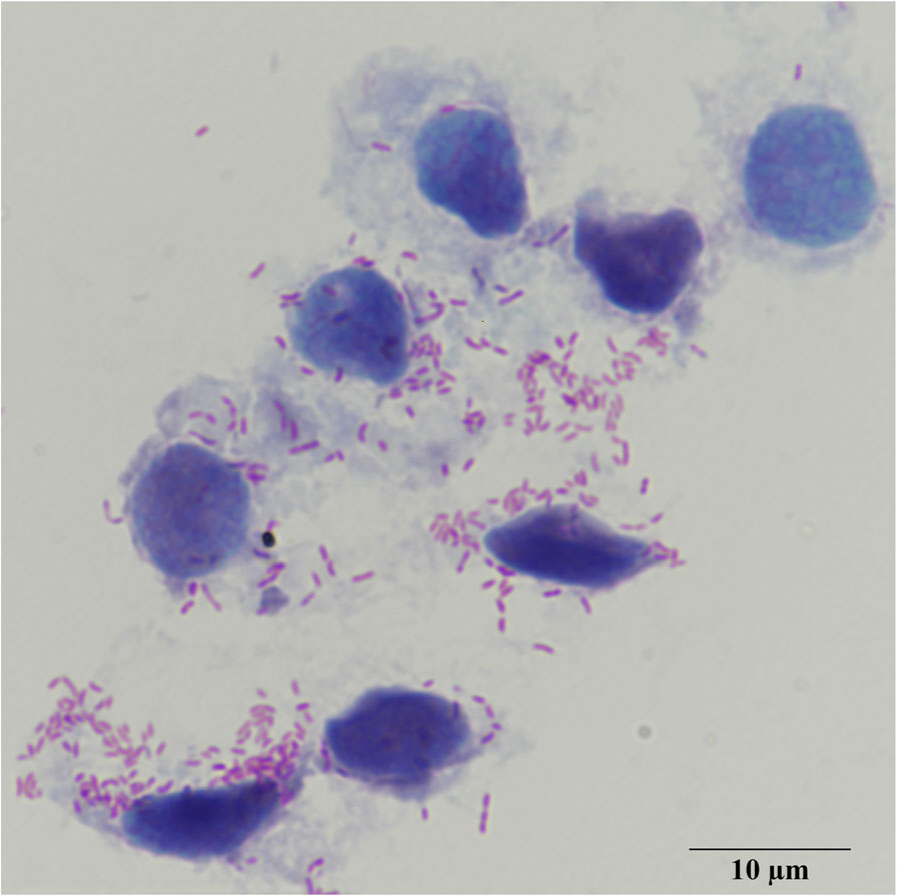
Infected tick cells, Giménez stain in which the bacteria are stained *red/pink* and the host cells *blue*

**Fig. 3:**
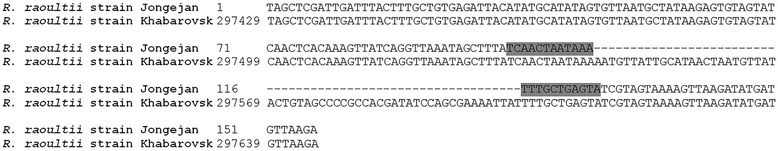
Alignment, obtained with MEGA version 6, of the 23S-5S ribosomal RNA intergenic spacer of *R. raoultii* strain Jongejan in comparison with *R. raoultii* strain Khabarovsk (CP010969.1), showing a 60 bp deletion in the intergenic spacer region. The *R. raoultii* strain Jongejan-specific probe sequence is highlighted in *grey*, spanning this deletion

We incubated cell suspensions of *D. reticulatus* with the tick cell line *R. microplus* BME/CTVM2, and after 65 days we observed intracellular bacteria in the cultured cells (Fig. 2). The negative control culture remained free of (intracellular) bacteria. On the basis of the microscopy we suspected that the intracellular bacteria were a species of *Rickettsia*; this was confirmed in several PCRs targeting different genes. PCR fragments sent for sequencing showed 99–100 % (KX500092–KX500097) identity with *R. raoultii* sequences available in the NCBI GenBank database (Table 2). Of note, we observed a 60 bp deletion when the 23S-5S ribosomal RNA intergenic spacer fragment was compared with the full genome sequence available for *R. raoultii* strain Khabarovsk (CP010969.1) (Fig. 3). A strain-specific RLB probe was designed to span this 60 bp deletion (Fig. 3). Furthermore, the obtained sequence of the *sca4* gene showed insertion of a single nucleotide that caused a frameshift resulting in a pseudogene. The isolated strain showed high identity with *R. raoultii* and we named this strain *R. raoultii* strain Jongejan.

**Table 2:** Relationship of *R. raoultii* strain Jongejan with available NCBI sequences

Target	Accession no.	Size (bp)	Identity (%)^a^	Closest related *R. raoultii* sequence
16S ribosomal RNA	KX500096	252	99	KJ410261.1
23S-5S ribosomal RNA intergenic spacer	KX500097	157	100^b^	CP010969.1
*sca4*	KX500095	802	100^c^	DQ365807.1
*ompA*	KX500093	513	100	AH009131.2
*ompB*	KX500094	710	100	KU310593.1
*gltA*	KX500092	259	100	KU310589.1

^a^Identity according to NCBI BLAST (blast.ncbi.nlm.nih.gov) analysis

^b^Contains a 60 base-pair deletion when compared with the reference genome (CP010969.1)

^c^Single nucleotide insertion results into a pseudogene

Viability of the newly isolated strain was assessed by (sub)inoculating either 100 μl cell suspension or 100 μl medium (taken without disturbing the cell layer). Both methods successfully infected BME/CTVM2 cells: identical intracellular bacteria were present in cytocentrifuge smears of these newly infected cultures within 14 to 21 days. We also used a PCR-RLB assay targeting the 23S-5S ribosomal RNA intergenic spacer and the newly designed strain-specific probe to confirm the successful subculture of *R. raoultii* strain Jongejan.

Cryopreservation and resuscitation of *R. raoultii* strain Jongejan proved successful as both, 80 °C and in liquid nitrogen stored cell suspensions successfully infected BME/CTVM2 cells; after 28 days identical intracellular bacteria were observed while analyzing the cytocentrifuge smears.

## Discussion

Previously, the centrifugation-shell vial technique described by Marrero & Raoult [32] was used to isolate *R. raoultii* in VERO and L929 mammalian cell line cultures [33]. However, the method required specialized equipment and more regular maintenance of the cell lines, making it laborious and less cost efficient. More recently, tick cells have been used to isolate *R. raoultii*, e.g. embryo-derived culture cell lines originating from *R. sanguineus* (RLM-RSE) [34] or embryo-derived primary cell lines of *D. reticulatus* [27]. After an initial cultivation period of 14–16 months in primary *D. reticulatus* cell lines, propagation of *R. raoultii* was successful in a number of tick cell lines originating from *D. albipictus*, *D. nitens*, and *R. microplus* [27], using the same *R. microplus* cell line as used in our study. It is possible that the initial cultivation period of 14–16 months aided the adjustment of *R. raoultii* towards the laboratory cultivation conditions, making subcultivations more likely to succeed. We show for the first time the capability of the *R. microplus* cell line BME/CTVM2 as initial isolation host for *R. raoultii*. Successful cryopreservation and resuscitation of *R. raoultii* has, to the best of our knowledge, not been documented before.

The sequences obtained from the newly named *R. raoultii* strain Jongejan showed differences within the 23S-5S ribosomal RNA intergenic spacer and the *sca4* gene when compared with the full genome sequence of strain Khabarovsk (CP010969.1). The 23S-5S spacer is non-coding, therefore selective pressure is low and mutations within the spacer can occur frequently. Due to the non-coding nature of the spacer, the deletion most likely does not influence the behavior or the pathogenicity of the bacteria. The *sca4* gene, together with the *ompA* and *ompB* genes, are part of a group coding the cell surface antigen proteins (immunodominant surface protein antigens) forming the outer cell surface of *Rickettsia* bacteria [35]. In patients, the immune response towards these proteins is strong [36], and the proteins are under positive selection pressure to evade the immune system, which results in high variation within these genes [35]. Furthermore, the single nucleotide insertion is also present within another published sequence of *R. raoultii* strain Marne (DQ365807.1); Strain Jongejan varies to strain Marne within the 16 s rRNA and *ompB* genes by 1 % (identity 99 %). The specific phenotypical change due to the *sca4* mutation needs to be further studied in order to assess the bacterial behavior and possible change to the pathogenicity of this strain. Considering the distinct molecular characteristics of strain Jongejan, we designed a specific probe for its detection in field specimens, thus enabling screening on a geographical basis and determination of the distribution of this strain.

Together with previous work [21, 27, 34], our study shows that tick cell lines are an aid in the isolation of tick-borne microorganisms, thus benefiting medicine and research. The use of such cell lines provides a platform for development of serological tests for screening patients and facilitates further study of the organisms. Future research should assess the capability of tick cell cultures in isolation of *Rickettsia* species from clinical samples. Our results also demonstrate that tick cell lines might be useful in culturing tick-borne pathogens that are currently uncultivable, such as *Candidatus* Neoehrlichia mikurensis. Moreover, geographical screening of *D. marginatus* and *D. reticulatus* would be beneficial in investigation of the distribution of different *R. raoultii* strains, both locally in Austria and in larger areas within Eurasia.

## Conclusions

During our study *R. raoultii* has been successfully isolated and propagated. Genetic analysis of the isolated strain revealed significant differences compared to the currently available data in the NCBI GenBank database. These molecular variations might lead to differences in phenotype and the behavior of the microorganism, potentially increasing the risk for human health. Furthermore our study shows for the first time the successful cryopreservation and resuscitation of *R. raoultii*.

## Abbreviations

DEBONEL: *Dermacentor*-borne necrosis erythema and lymphadenopathy
DMSO: Dimethylsulfoxide
RLB: Reverse line blot
TIBOLA: Tick-borne lymphadenopathy
